# Ab initio study of structural, optoelectronic, magnetic, and mechanical properties of Cs_2_KXBr_6_ (X = Mo, W): HDPs for spintronic and energy applications

**DOI:** 10.1038/s41598-026-56062-y

**Published:** 2026-06-02

**Authors:** Ahmad Ali, Muhammad Hashir, Danyal Khan, Sikander Azam, Asif Nawaz Khan, Hijaz Ahmad

**Affiliations:** 1 Department of Physics, Government Degree College Lahor, Swabi, Pakistan; 2 Faculty of Basic Sciences, Ghulam Ishaq Khan Institute of Engineering Sciences and Technology, Topi, 23460 Pakistan; 3 Department of Physics and Mathematics, University of Hull, Hull, HU6 7RX UK; 4 New Technologies – Research Centre, University of West Bohemia, 8 Univerzitní, 306 14 Plzeň, Czech Republic; 5 Materials Modeling and Simulation Lab, Department of Physics, University of Science and Technology, Bannu, 28100, Khyber Pakhtunkhwa Pakistan; 6 Irfan Suat Gunsel Operational Research Institute, Near East University, 99138 Mersin 10, Nicosia, TRNC Turkey; 7 Department of Software Engineering, Karadeniz Technical University, Trabzon, 61080 Turkey; 8 VIZJA University, Okopowa 59, 01-043 Warsaw, Poland; 9 Faculty of Engineering and Applied Sciences, Department of Physics,, Riphah International University, Islamabad, Pakistan; 10 Department of Technical Sciences, Western Caspian University, Baku, 1001 Azerbaijan; 11 Department of Mathematics, College of Science, Korea University, 145 Anam-ro, Seongbuk-gu, Seoul, 02841 South Korea

**Keywords:** DFT calculations, Double halide perovskites, Structural properties, Optoelectronic properties, Magnetic properties, Materials science, Physics

## Abstract

The structural, optoelectronic, magnetic, and mechanical investigations of Cs_2_KXBr_6_ (X = Mo & W); halide double perovskites (HDPs), in the Fm-3m space group, have been conducted via an ab initio study. The formation energies per atom (eV) of Cs_2_KMoBr_6_ and Cs_2_KWBr_6_ are found to be − 1.72 and − 1.81, respectively. To reveal the accurate energy band gaps of the materials, the TB-mBJ potential is utilized. The electronic analysis reveals that both materials exhibit semiconducting behavior in both spin channels. The energy band gaps of Cs_2_KMoBr_6_ are 1.7 eV (up-spin) and 2.8 eV (down-spin), and Cs_2_KWBr_6_ are 1.6 eV (up-spin) and 3.3 eV (down-spin). The Phonon dispersion analysis and ab initio* molecular dynamics* simulations reveal the dynamical stability of the materials. The ferromagnetic (FM) and antiferromagnetic (AFM) natures of Cs_2_KWBr_6_ and Cs_2_KMoBr_6_ have been predicted, respectively. The net magnetic moment (µ_B_) of the ground state of Cs_2_KWBr_6_ and Cs_2_KMoBr_6_ is 3 and 0, respectively. The mechanical analysis reveals that Cs_2_KWBr_6_ exhibits higher structural stability compared to the Cs_2_KMoBr_6_ material. The computed optical properties indicate that the materials have high absorption with a maximum of 140 cm^−1^ at 13.5 eV, a peak refractive index of 1.9 between 4.5 and 5.8 eV, and a maximum reflectivity of about 0.40 at high energy, which makes them promising candidates for high-energy region and moderate refractive indices, suggesting potential relevance for optoelectronic applications. In addition, the spin-dependent electronic structure and magnetic ordering indicate possible applicability in spin-dependent electronic systems. However, further device-level investigations are required to fully assess their technological performance.

## Introduction

The increasing tendency to develop new compounds that possess improved physical qualities has provided bright perspectives in future technological developments. Consequently, a lot of effort has been made by material scientists to design and develop novel compounds with enhanced functional behavior. These are new materials that have drawn a lot of theoretical and experimental attention because of their broad applicability in industry and technology. It is important to note that they are involved in spintronic, magnetic optical, half-metallicity, thermoelectric, ferromagnetism, magneto-dielectrics, semiconductors, and magneto-electronic equipment in solid-state technology^1–4^. In recent years, there has been significant interest in rare earth and alkaline earth materials. Perovskites categorized as transition-metal perovskites represent a specific subset within the broad classification of double perovskites^5,6^. The general formula of a double halide perovskite is A_2_BB′X_6_, which comprises a monovalent A-site cation, B and B′ cations in the + 1 and + 3 oxidation states, and the halide ions at the X sites, which provides broad structural and electronic tunability with application in advanced devices^7^. Materials scientists and physicists have been paying more attention to perovskites because of their compositional versatility, low-cost synthesis, and impressive functional characteristics. In particular, double perovskites (DPs) have gained prominence recently because of their diverse physical characteristics that are appealing from a technology perspective^8,9^. Theoretical analysis utilizing ab initio quantum computations has emerged as an important method for revealing the relationship between crystal structure and optical performance. These correlations provide the most effective production in various optoelectronic materials and significantly benefit in the interpretation of experimental data.

Nabi et al.^10^ have reported the Cs_2_GeVX_6_ (X = Cl, Br, I) double halide perovskites using first principles studies. The materials were found to be appropriate for both visible and ultraviolet optoelectronic applications due to their bandgap of 2–3 eV. Shafiq et al.^11^ have assessed the structural, mechanical, optical, and thermoelectric properties of Cs_2_GeSnX_6_ (X = Cl, Br) employing the DFT. The results indicate that the materials could be used in thermoelectric and optoelectronic applications. Liu et al.^12^ have studied the structural, thermodynamic, mechanical, electrical, and optical characteristics of Cs_2_B′BiI_6_ (B′ = Li, Na, and K) materials via DFT. Dar et al.^13^ employed a first-principles study to investigate the physical properties of Cs_2_ScInBr_6_, a halide double perovskite. The analysis of optical properties indicates that the material is suitable for use in infrared devices. The magnetic, optoelectronic, and transport properties of Cs_2_XMoBr_6_ (X = Na, Li) have been studied by Zia Ullah et al.^14^. McClure et al*.*^15^ investigated the lead-free double halide perovskites Cs_2_AgBiX_6_ (X = Cl, Br) through theoretical and experimental means. The materials are found to be promising for photovoltaic applications. The stability of the inorganic halide DP family Cs_2_CuMCl_6_ (M = Sb, Bi) in the cubic phase and their potential as optoelectronic candidates have been predicted by Nabi et al*.*^1^. Obada et al.^16^ have conducted a review on a large number of double halide perovskites by studying their structural, optical, thermoelectric, and mechanical properties. The materials are found to have a wide band gap semiconducting nature. The review aims to identify novel perovskite materials and provide a thorough evaluation. Ali et al*.*^17^ have recently reported the Cs_2_XMoI_6_ (X = K, Li, and Na) halide double perovskites by investigating their optoelectronic and magnetic properties. Caid et al.^18^ have explored the structural stability and optoelectronic properties of Cs_2_B′B″Br_6_ (B′B″: BeMg, CdBe, CdGe, GeMg, GeZn, MgZn) double halide perovskite materials, using ab initio investigations. The materials are found to be semiconducting in nature. Detailed and comprehensive first-principles investigations on Cs_2_B′GeCl_6_ (B′: Zn, Cd) have been conducted to check their suitability for optoelectronic and other affordable energy technologies^19^. Amika et al.^20^ employed a twofold method, which is a combination of hydrothermal synthesis and density functional theory (DFT) computations to study PbS-doped bismuth titanate. Their findings show that the narrow-bandgap PbS doping is an effective way to sensitize the perovskite to absorb visible light and enhance the separation of charge carriers.

While related double perovskites such as Cs_2_XMoBr_6_ (X = Na, Li) have been previously investigated by Bani-Fwaz et al.^21^, the present study introduces K substitution at the A-site, which significantly modifies the structural environment and electronic interactions due to its larger ionic radius. Unlike Na and Li, the presence of K leads to subtle lattice expansion and altered metal–halide bonding characteristics, which directly influence the electronic bandwidth and magnetic exchange interactions. Furthermore, the comparative analysis between Mo (4*d*) and W (5*d*) based systems provides deeper insight into the role of *d*-orbital extension and hybridization strength in governing spin-dependent electronic properties. In particular, the interplay between crystal-field effects, exchange splitting, and *d*–*p* hybridization results in pronounced differences in band gaps, magnetic ordering, and mechanical stability. Therefore, this work not only extends the compositional space of Cs_2_KXBr_6_ (X = Mo & W); HDPs, but also provides a systematic understanding of how A-site substitution and transition-metal selection influence the electronic and magnetic behavior of halide double perovskites. The literature review confirms the novelty of the materials, indicating that no experimental or theoretical research has been conducted on these materials to date.

## Computational method

Three density functional theory (DFT)-based computational codes, including Wein2k, Quantum Espresso, and CASTEP, were used to investigate the properties of the materials. The WIEN2k is used to compute high-precision electronic properties using the Full Potential Linearized Augmented Plane Wave (FP-LAPW) method, and Quantum Espresso and CASTEP were used to compute structural relaxation and dynamical stability problems efficiently. It has been ensured that structural parameters are very similar across all three platforms, so that the results are robust and do not depend on the computational framework. The Wein2k computational code is used to perform the spin-based first principles investigations, by computing the structural, optoelectronic, and magnetic characteristics of Cs_2_KXBr_6_ (X = Mo & W); HDP’s materials^22^. The FP-LAPW method has been used, where the plane wave solution in the interstitial region and the spherical harmonic wave function inside the core must be used to solve the wave function^23^. The structural calculations were used to optimize the system’s ground state energy and lattice characteristics. To enhance the precision of band gap computations in electronic systems, researchers employed a modified Becke–Johnson (mBJ) potential combined with the PBE-GGA approximation. The fundamental analytical form of the exchange potential is considered to be the modified Becke–Johnson potential^24^. The Trans-Blaha-modified Becke–Johnson (TB-mBJ) method was chosen for this investigation because of its track record of precisely estimating the band gap in close approximation to experimental values^25^. The Becke–Johnson potentials are employed to enhance the energy band gap difference, calculated using density functional theory.

1 $$\upsilon_{\chi , \sigma }^{TB{\mathrm{-}}mBJ} \left( r \right) = c\upsilon_{\chi , \sigma }^{BR} \left( r \right) + \left( {3c - 2} \right) \frac{1}{\pi }\sqrt{\frac{5}{6}} \sqrt {\frac{{\tau_{\sigma } \left( r \right)}}{{\rho_{\sigma } \left( r \right)}}}$$

The constraint limits the TB-mBj of determining total energies and Hellmann–Feynman coefficients.

Self-consistency was achieved by employing an angular momentum cutoff of l_max_ = 10 in the muffin-tin spheres and G_max_ = 12 in the interstitial region. A 10 × 10 × 10 k-point mesh (1000 k-points) was used to sample the first Brillouin zone. To ensure the numerical accuracy of the calculations, convergence of the total energy was carefully tested with respect to k-point sampling and basis-set size. It was verified that increasing the k-point density beyond the adopted grid results in negligible changes in total energy and electronic structure. The plane-wave cutoff parameter was controlled through the R_MT_ × K_MAX_ value, which was set to 7, where R_MT_ is the smallest muffin-tin radius and K_MAX_ is the maximum plane-wave vector. Further increases in this parameter produced only marginal variations in total energy, confirming the adequacy of the chosen basis set. The cutoff energy was fixed at − 6.0 Ry. Energy and charge convergence were achieved iteratively through self-consistent cycles until the differences between successive iterations were less than 10^−4^ Ry for total energy and 10^−3^ e for charge density. These criteria ensure well-converged and numerically reliable results for all calculated structural, electronic, and magnetic properties. The muffin-tin radius RMT is calculated to be 2.50, 2.50, 2.34, 2.39, and 2.34 for Cs, K, Mo, W, and Br atoms, respectively. To ensure accuracy, the valence states in the electronic structure computations were designed to include low-lying semi-core states; the configurations used were 5*s*^2^, 5*p*^6^, 6*s*^1^ for Cs, 3*s*^2^, 3*p*^6^, 4*s*^1^ for K, 4*s*^2^, 4*p*^5^ for Br, 4*s*^2^, 4*p*^6^, 4*d*^5^, 5*s*^1^ for Mo, 5*s*^2^, 5*p*^6^, 5*d*^4^, 6*s*^2^ for W, and 2*s*^2^, 2*p*^4^ for O. The 1000 k-points were employed to compute the density of states and optical properties of the materials. The materials’ optical properties are described by their frequency-dependent dielectric function, ε(ω)^26^, which has been given by the relation;

2 $$\upvarepsilon \left(\upomega \right) =\upvarepsilon _{1} \left(\upomega \right) + {\mathrm{i}}\upvarepsilon _{2} \left(\upomega \right)$$

Optical transitions happen when photon and electron fields of light interact. The imaginary portion of the dielectric function ε_2_(ω) has been found by the momentum matrix dipole. This has been characterized by the Kramer–Kronig relations, and is presented as^27,28^,

3 $$\varepsilon_{1} \left( \omega \right) = 1 + \frac{2}{\pi }P\mathop \int \limits_{0}^{\infty } \frac{{\omega \varepsilon_{2} \left( \omega \right)}}{{\omega ^{{\prime}2} - \omega^{2} }}d\omega^{\prime}$$

4 $$\varepsilon_{2} \left( \omega \right) = \frac{{e^{2} { }\hbar }}{{\pi m^{2} \omega^{2} }}\mathop \sum \limits_{v,c} \int BZ|M_{cv} \left( k \right)^{2} \delta \left[\omega_{cv} \left( k \right) - \omega \right]d^{3} k$$

The imaginary component of the dielectric function is responsible for interband transitions from the valence band (occupied states) to the conduction band (unoccupied states). The ab initio* molecular dynamics* (AIMD) simulations are carried out for both materials, using the Quantum Espresso computational code^29,30^. The AIMD simulations were conducted in a primitive unit cell with 10 atoms and displayed in Fig. 3(a and b). A 7 × 7 × 7 Monkhorst–Pack k-point mesh was used to sample the Brillouin zone. Simulations were run at time steps of 0.966 fs and a total of 7000 time steps. Ultra-soft pseudopotentials were used with a plane-wave kinetic energy cutoff of 65 Ry and charge-density cutoff of 350 Ry. The velocity-Verlet algorithm in the micro-canonical (NVE) ensemble was used to evolve the ionic motion, and the system was free to evolve, without any external thermostats. The *Cambridge Sequential Total Energy Package* (CASTEP) code^31^ was used to compute the mechanical properties in the framework of density functional theory (DFT). The optimized crystal structure was subjected to finite deformations in order to get the elastic constants using the stress–strain method. The Voigt–Reuss–Hill (VRH) approximation was used to determine the bulk modulus, shear modulus, Young’s modulus, and Poisson’s ratio.

## Result and discussion

### Structural properties

**Fig. 1:**
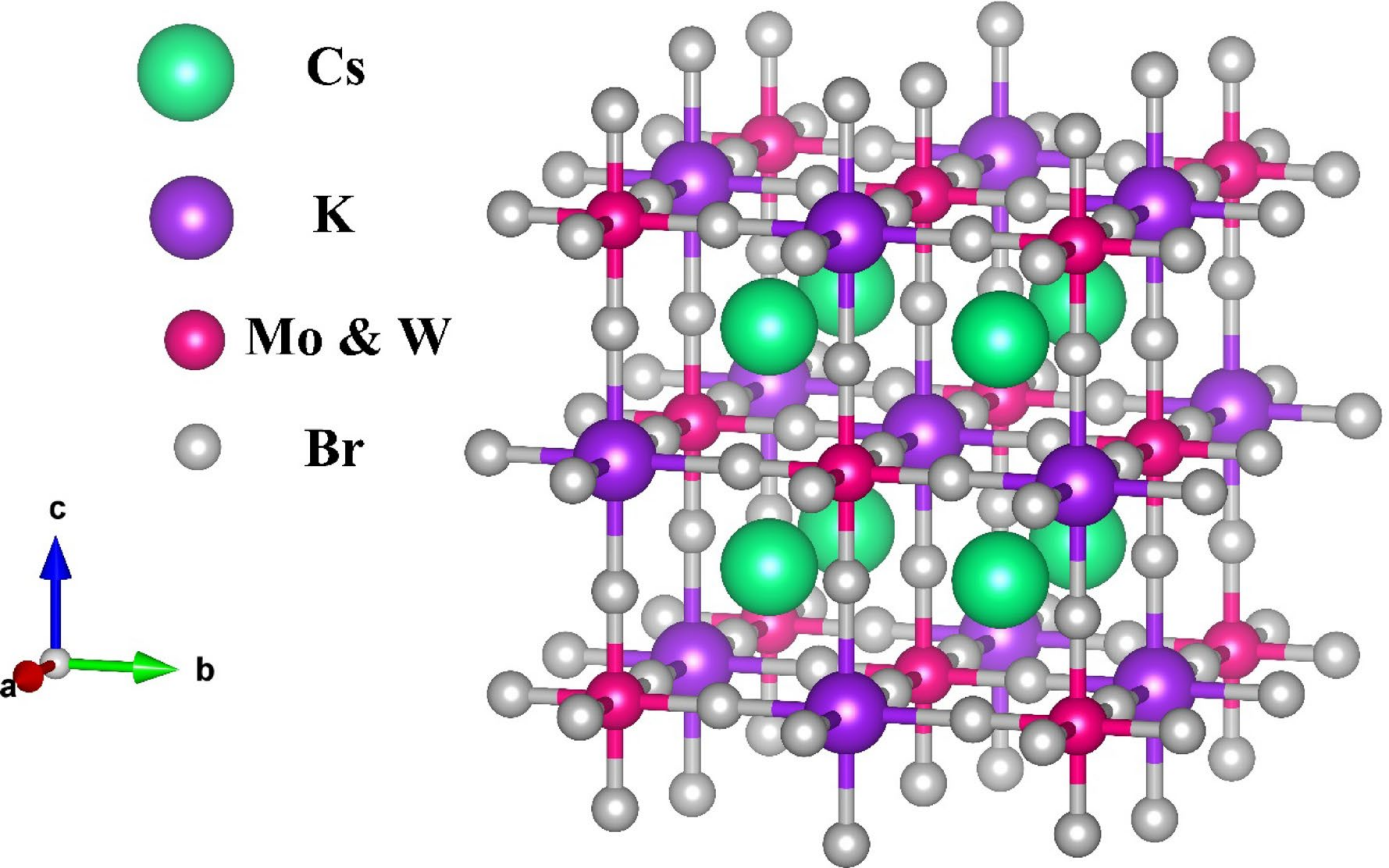
The structure of Cs_2_KXBr_6_ (X = Mo & W); HDPs in cubic phase. By Vesta Code.

**Fig. 2:**
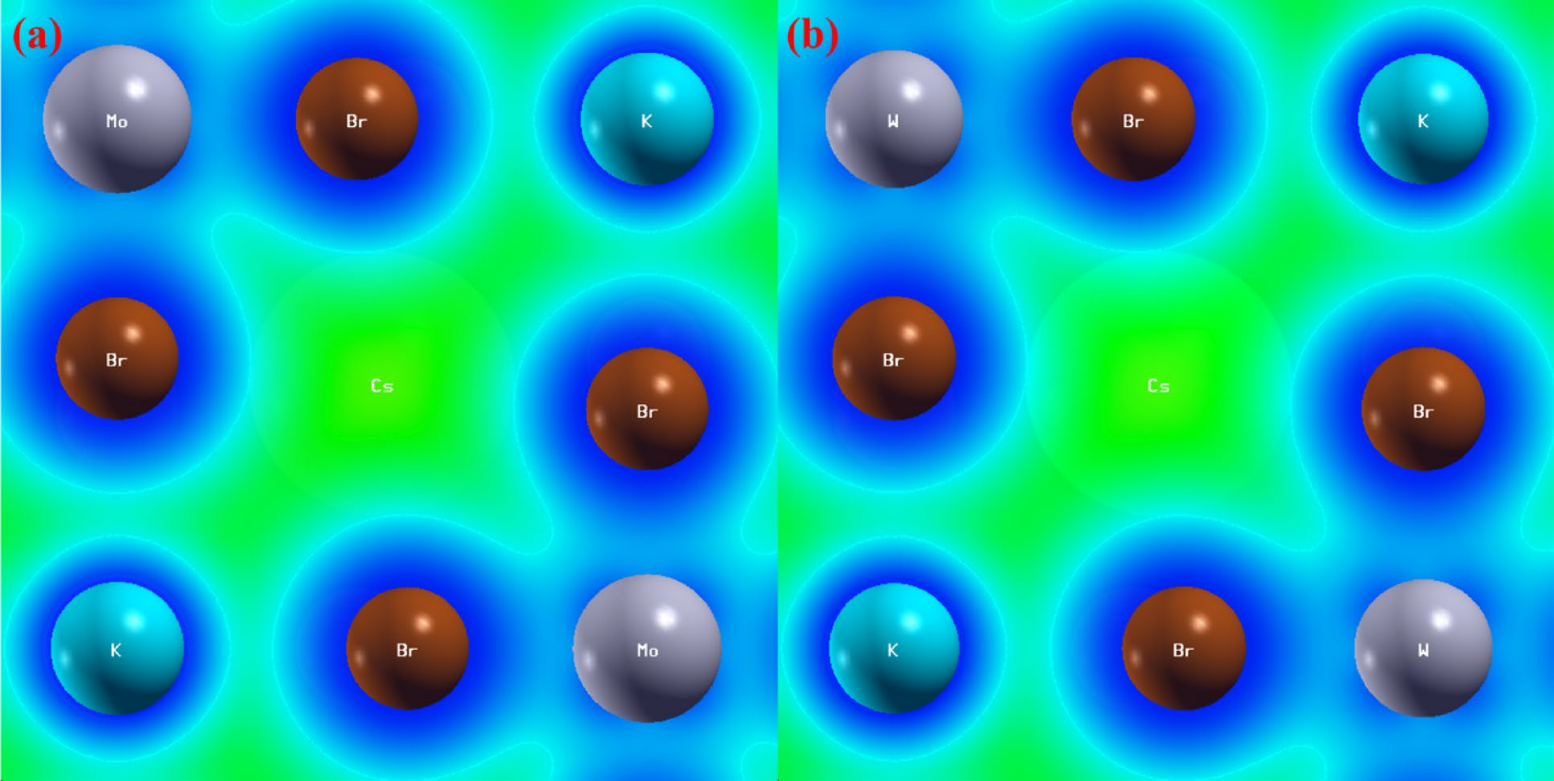
The electron charge densities of (**a**) Cs_2_KMoBr_6_ and (**b**) Cs_2_KWBr_6_ perovskite materials.

The Cs_2_KMoBr_6_ and Cs_2_KWBr_6_ materials crystal structures in the cubic Fm-3m space group, displayed in Fig. 1, are optimized by using energy vs volume optimizations (see Fig. 7). The optimized lattice constants, crystallographic volume, bulk modulus, ground state energies, formation energy per atom (eV), and tolerance factor, listed in Table 1, are the important optimized structural parameters of the materials. The predicted volume of Cs_2_KMoBr_6_ is larger than Cs_2_KWBr_6_ volume because stronger W–Br bonding and relativistic effects in W^6+^ lead to lattice contraction despite its slightly larger ionic radius. The computed Bulk modulus of the Cs_2_KMoBr_6_ and Cs_2_KWBr_6_ are 22.14 GPa and 23.68 GPa, respectively. The higher bulk modulus of Cs_2_KWBr_6_ compared to Cs_2_KMoBr_6_ can also be attributed to the stronger W–Br bonds. The reason behind stronger W–Br bonds can further be verified by charge density analysis. The electronic charge densities of both materials are computed, depicted in Fig. 2(a and b), to analyze the atomic bond interactions. The plots reveal stronger W–Br hybridization compared to the more localized Mo–Br bonding, which results in lattice contraction and a higher bulk modulus of Cs_2_KWBr_6_ compared to Cs_2_KMoBr_6_, demonstrating bond stiffness.

**Table 1:** The structural parameters of Cs_2_KXBr_6_ (X = Mo & W) HDP’s materials.

Structural parameters	Cs_2_KMoBr_6_	Cs_2_KWBr_6_
Space group	Fm-3m (225)	Fm-3m (225)
Optimized lattice constants (Å)	22.916464	22.96263
Volume (a.u^3^)	2619.1203	2604.7767
Bulk modulus (GPa)	22.14	23.68
Ground state energy; E_0_ (Ry)	− 71,745.25939	− 95,978.272823
Formation energy/atom; ΔH_F_ (eV)	− 1.72	− 1.81
Tolerance factor (τ)	1.032	1.027

### Structural stabilities

The assessment of structural stability is a fundamental prerequisite for determining the experimental feasibility and potential synthesis of the investigated materials. The prediction of formation energy aids in comprehending the materials’ thermodynamic stability. The formation energy ΔH_F_ of the Cs_2_KXBr_6_ (X = Mo & W) materials can be computed by utilizing the following relation;

5 $$\Delta H_{F} = \frac{{E_{{(Cs_{2} KXBr_{6} \left( {X = Mo/W} \right)}} - \left( {2E_{Cs} + E_{K} + E_{X = Mo/W} + 6E_{Br} } \right)}}{10}$$

The relation contains E_Cs2KXBr6 (X=Mo W)_; the total energy of the compounds, and E_Cs,_ E_K_, E_Mo_, E_W_, and E_Br_ represent the energies of Cs, K, Mo, W, and Br elements, respectively, computed by energy optimizations analysis, displayed in Fig. 7, in their lowest energy phases. The computed formation energies per atom in eV, of Cs_2_KMoBr_6_ and Cs_2_KWBr_6_ are − 1.72 and − 1.81, respectively. The negative formation energies predict the thermodynamic stability of both materials. The structural stability of a compound can be assessed by analyzing various stability criteria applicable to the materials involved. The tolerance factor τ, as defined by Goldschmidt in a modified format, serves as an important factor for determining the phase stability of the materials under investigation, and is defined as^32^;

6 $$\tau = 0.707 \times \frac{{r_{Cs} + r_{Br} }}{{\left( {\frac{{r_{K} + r_{Mo/W} }}{2} + r_{Br} } \right)}}$$

Here, the r_Cs_, r_K_, r_Mo_, r_W_, and r_Br_ represent the ionic radii of the elements Cs, K, Mo, W, and O, respectively. The ionic radii of each element are suggested by Shannon et al.^33^. The calculated tolerance factors (~ 1.03) of both materials, in the cubic structure, fall within the permitted stable range (0.825 *< τ < 1.059)* for halide double perovskites^34,35^.

**Fig. 3:**
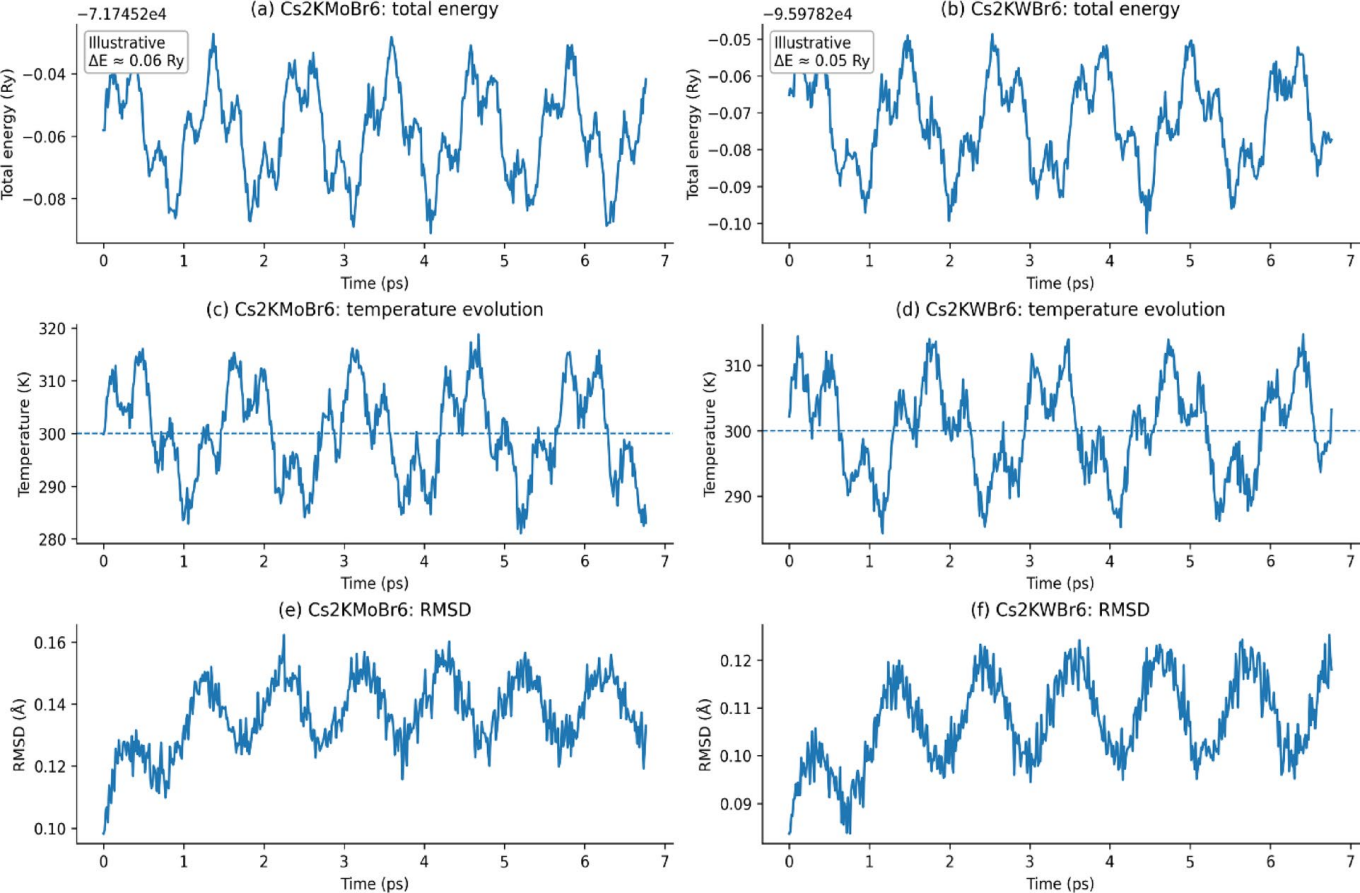
AIMD-based thermal-stability analysis of Cs_2_KXBr_6_ (X = Mo, W) at 300 K: (**a**, **b**) total energy as a function of simulation time, (**c**, **d**) temperature evolution, and (**e**, **f**) RMSD relative to the initial structure. The absence of strong thermal drift and the small RMSD oscillations indicate short-time structural stability during the simulated time window.

To further assess the thermal stability of Cs_2_KXBr_6_ (X = Mo, W), we expanded the AIMD analysis by explicitly reporting the simulation temperature and by including additional structural stability descriptors beyond total-energy variation. The AIMD simulations were carried out for approximately 6.8 ps, and the temperature evolution was monitored throughout the trajectory. As shown in Fig. 3c and d, the instantaneous temperature fluctuates around the target value of 300 K without any systematic drift, which indicates stable thermal behavior during the simulated time window. Such fluctuations are expected for a finite system in molecular dynamics and do not suggest structural degradation^36^.

In addition to the total energy, the root-mean-square deviation (RMSD) was evaluated as a function of time to quantify structural deviations from the initial configuration. The RMSD profiles of both Cs_2_KMoBr_6_ and Cs_2_KWBr_6_, shown in Fig. 3(e and f), exhibit only small oscillatory variations and quickly reach a nearly steady regime, with no abrupt increase that would indicate bond breaking, atomic diffusion, or framework collapse. The total-energy traces in Fig. 3(a and b) also remain bounded within a narrow fluctuation range during the entire simulation. Taken together, the stable energy evolution, the absence of temperature drift, and the small RMSD values provide consistent evidence that both compounds preserve their structural integrity during AIMD at 300 K.

At the same time, we note that the present AIMD time scale is limited to the short-picosecond regime. Therefore, these results should be interpreted as evidence of short-time thermal stability at room temperature, rather than as a definitive proof of long-time thermal robustness under all operating conditions. In this sense, the revised AIMD analysis substantially strengthens the stability discussion while maintaining an appropriately cautious interpretation.

**Fig. 4:**
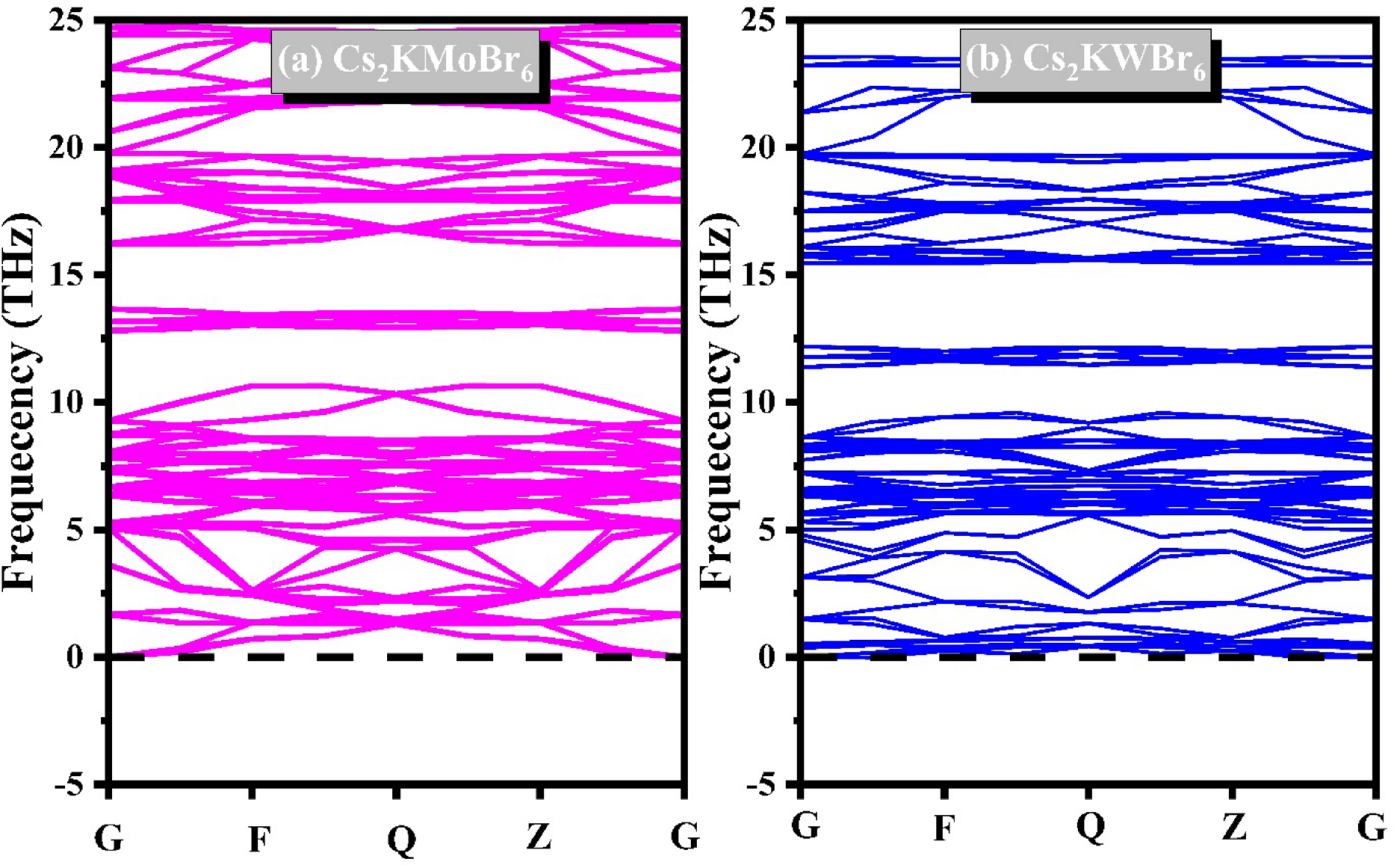
The phonon dispersion of (**a**) Cs_2_KMoBr_6_ and **(b)** Cs_2_KMoBr_6_ perovskite materials.

The phonon dispersion curves can give valuable information about the dynamical stability and the vibrational behavior of the lattice of the Cs_2_KMoBr_6_ and Cs_2_KWBr_6_ materials, depicted in Fig. 4(a and b). The plots of the dispersions are computed along the high-symmetry directions (G-F-Q-Z-G) in the Brillouin zone. Throughout the high-symmetry Brillouin zone, all phonon branches in both compounds remain positive, and no imaginary frequencies are noticed. The lowest-frequency modes with their origin at the G-point in the phonon dispersion plots are the acoustic ones to which the 0 Hz frequency is connected, and then extending up to approximately 5 THz. The optical branches are found at higher frequencies, beginning with finite values at the G point and rising all the way to about 25 THz. These are more numerous and relatively flatter than the acoustic branches, which are indicative of localized vibrations in the unit cell. The occurrence of discrete acoustic modes in Cs_2_KMoBr_6_ and Cs_2_KWBr_6_ with no imaginary frequency, which imply that both structures are stable elastically, proves that the crystal structures are dynamically stable.

### Electronic properties

**Fig. 5:**
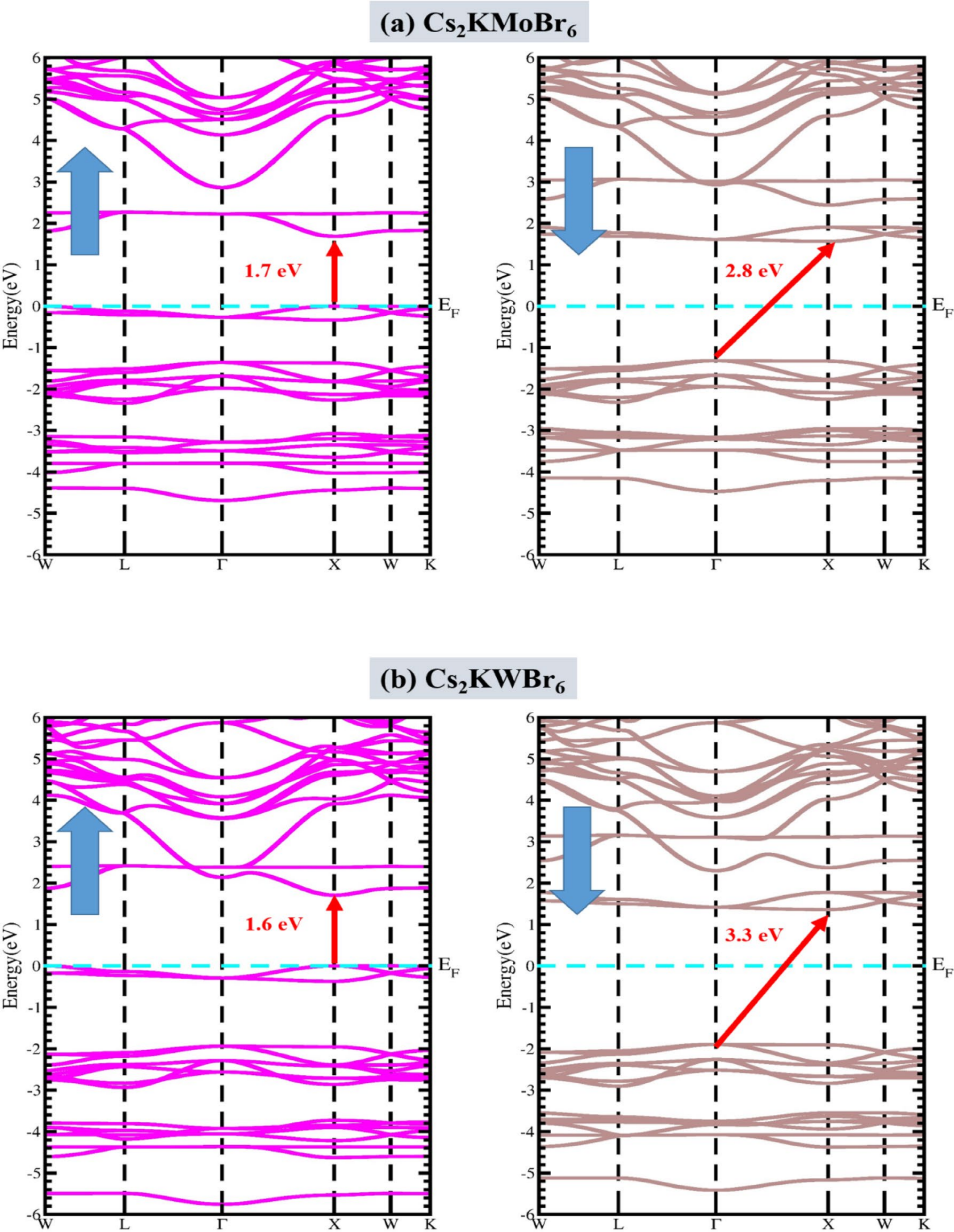
The spin-polarized electronic band structures of (**a**) Cs_2_KMoBr_6_ and (**b**) Cs_2_KWBr_6_ perovskite materials.

The electronic properties deal with the effect of electric fields, charge carriers, and voltage seams on a material’s electrical properties. In order to use a material effectively in optoelectronics, electronics, or energy device applications, one must learn about its electrical characteristics^37–39^. The materials under examination are assessed for their electrical properties, specifically their band structure, to identify whether they are metals, semiconductors, or insulators. Materials including metals, semiconductors, and insulators can be categorized based on their carrier concentrations, band gap values, and carrier mobilities^40,41^. The energy band gap of a material can be determined by the energy gap between the conduction band minimum (CBM) and valence band maximum (VBM). The energy bands are separated by the Fermi level (E_F_), which has been set at 0 eV. Fig. 5(a and b) displays the computed electronic band structures of the materials, in both spin configurations. The energy band gap of Cs_2_KMoBr_6_ is 1.7 eV (up-spin) and 2.8 eV (down-spin), and of Cs_2_KWBr_6_ is 1.6 eV (up-spin) and 3.3 eV (down-spin). The energy band gaps of the materials are compared with previous work done in the Table 2. The plots indicate that VBM and the CBM of these band structures do not lie at the same symmetrical point, revealing an indirect band gap semiconducting nature of the materials^42^. The well-defined energy band gaps in both spin configurations indicate a spin-dependent semiconducting nature of both materials.

**Table 2:** Comparison of the energy band gaps obtained in the present study with those reported in previous work on related materials.

Compounds	Up-spin		Down-spin		Nature of bands
	Present study (eV)	Related study^21^	Present study (eV)	Related study^21^	
Cs_2_KMoBr_6_	1.7	2.19 eV	2.8	2.66 eV	Indirect
Cs_2_KWBr_6_	1.6	–	3.3	–	Indirect

**Fig. 6:**
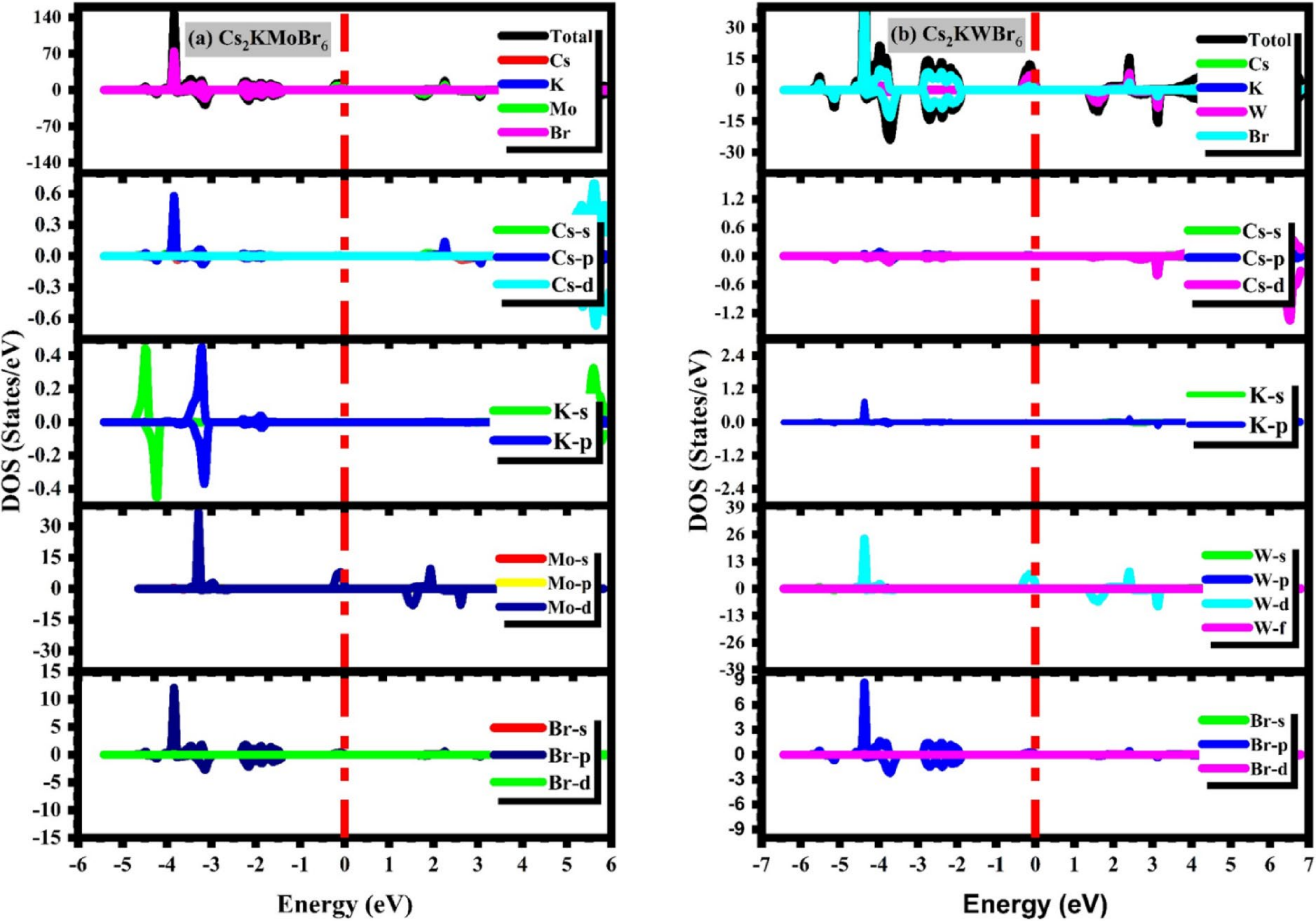
The spin-polarized density of states (DOS) of (**a**) Cs_2_KMoBr_6_ and (**b**) Cs_2_KWBr_6_ double perovskites.

Now, the focus of the study switches to the density of states (DOS) analysis to comprehend the role of electronic states in the electronic properties of the materials, explained by the electronic band structure. The computed spin-polarized DOS of both materials has been displayed in Fig. 6(a and b). The DOS suggests that the VBM of Cs_2_KMoBr_6_, in up-spin, is formed by K-3*p* and Mo-4*d* states, and by Br-3*d*, in down-spin, while the CBM is formed by K-3*p* and Mo-4*d* states, in both spin configurations. Similarly, the VBM of Cs_2_KWBr_6_, in up-spin, is formed by W-5*d* and in down-spin, by Br-4*p* state, and the CBM, in both spins, is formed by W-5*d* states. The DOS also demonstrates that the valence band of Cs_2_KMoBr_6_, in up-spin, is formed from 0 to − 5.8 eV, by the strong hybridizations of K-3*p* and Mo-4*d* states, and in down-spin, from − 1.8 to − 5 eV energy range, by the weak hybridizations of K-3*p*, Br-3*d*, and Cs-5*p* orbitals. Similarly, for Cs_2_KWBr_6_, the valence band, in up-spin, is formed from 0 to − 5.7 eV, due to W-5*d* and Br-4*p* states hybridizations, and the similar hybridizations can be seen, in down-spin, from − 2 to − 5.5 eV energy range. These atomic states interact strongly, resulting in considerable contributions of both elements near the Fermi level. In the down-spin configuration, the valence band is formed by Br-4*p* states of both materials. The electronic and magnetic nature of Cs_2_KMoBr_6_ and Cs_2_KWBr_6_ materials depends upon the Mo-4*d* and W-5*d* states, respectively. These states dominate the density of states near the Fermi level and control charge transport and magnetic exchange interactions. In contrast, instead of directly controlling the electrical or magnetic response, the Br orbitals indirectly influence lattice bonding, structural stability, and bandwidth modulation through hybridization with the metal states. The electronic charge density plots, Fig. 2, reveal that both materials exhibit a mixed ionic covalent bonding nature. The plots indicate the ionic interaction between Cs^+^ and K^+^ cations, while covalent bonding, due to significant orbital hybridization, occurs among Mo and W octahedra.

The large spin-dependent band-gap differences observed in Cs_2_KXBr_6_ (X = Mo, W) originate from the strong exchange splitting of the transition-metal d states, which is governed by the combined effects of crystal-field splitting, Hund’s exchange interaction, and hybridization with the surrounding Br ligands. In the cubic octahedral environment, the Mo^4+^ (4*d*^2^) and W^4+^ (5*d*^2^) ions experience a crystal-field splitting of the d orbitals into lower-energy t_2g_ and higher-energy e_g_ states. The partially filled t_2g_ manifold leads to spin polarization driven by intra-atomic exchange interactions (Hund’s rule), resulting in an energy separation between spin-up and spin-down channels.

The magnitude of this exchange splitting is further influenced by the spatial extension of the *d* orbitals. In particular, the more delocalized 5*d* orbitals of W exhibit stronger hybridization with Br-*p* states compared to the relatively localized Mo-4*d* orbitals. This enhanced hybridization increases bandwidth and modifies the exchange interaction strength, leading to a larger asymmetry between spin channels, especially in the conduction band. Consequently, the conduction-band minimum shifts differently in the two spin channels, producing the pronounced difference in spin-dependent band gaps (e.g., 1.6 eV vs 3.3 eV in Cs_2_KWBr_6_).

Furthermore, the indirect nature of the band gap amplifies this effect, since the valence-band maximum and conduction-band minimum arise from different k-points and orbital characters. As a result, even moderate exchange splitting at the atomic level can translate into a significant difference in the effective band gaps of the two spin channels. These findings indicate that the observed spin-dependent band-gap variation is not merely a numerical artifact, but rather a consequence of the interplay between crystal-field effects, exchange interactions, and orbital hybridization in these transition-metal-based double perovskites. The asymmetry between spin-up and spin-down DOS near the Fermi level directly reflects exchange-split *d* states, which are responsible for the observed spin-dependent band gaps.

Compared to previously reported Cs_2_XMoBr_6_ (X = Na, Li) systems, the substitution of K at the A-site introduces a larger ionic radius, which affects the lattice geometry and bonding environment. This modification leads to subtle changes in lattice constants and bond lengths, which in turn influence the electronic bandwidth and hybridization strength. As a result, the K-based systems exhibit distinct electronic and magnetic characteristics, including modified band-gap values and exchange interactions.

In addition, the comparison between Mo and W highlights the role of 4*d* versus 5*d* orbitals. The more spatially extended W-5*d* orbitals enhance hybridization with Br-*p* states, resulting in stronger bandwidth dispersion and more pronounced spin-dependent electronic behavior compared to the Mo-based counterpart. This demonstrates that both A-site substitution and transition-metal selection are key tuning parameters for controlling the physical properties of these materials.

### Magnetic properties

**Fig. 7:**
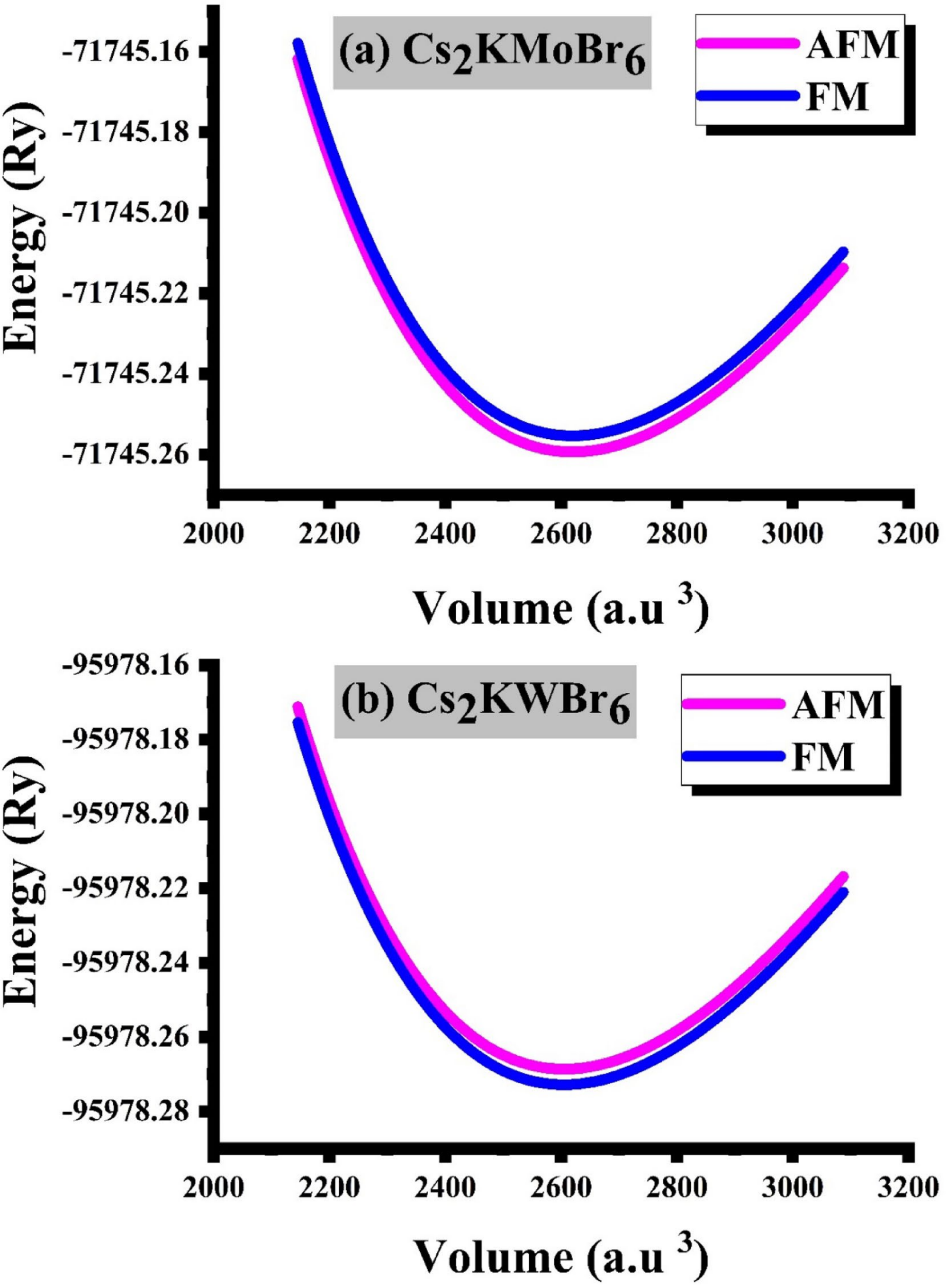
Total-energy comparison between FM and AFM configurations used to identify the magnetic ground state of (**a**) Cs_2_KMoBr_6_ and (**b**) Cs_2_KWBr_6_ perovskite materials.

The magnetic properties of double perovskites, which include ferrimagnetism, anti-ferromagnetism, and ferromagnetism, make them essential for spintronic, magneto-optical systems, and magnetic data storage^43^. In addition to providing insight into basic magnetic processes, studies of the magnetic properties of double perovskites may lead to the development of novel magnetic materials with special properties and potential applications in energy conversion, communication, and information storage^44^. The magnetic behavior of Cs_2_KXBr_6_ (X = Mo, W) was investigated using spin-polarized first-principles calculations to determine both the origin of magnetism and the magnetic ground state of each compound. To avoid ambiguity, it is important to distinguish between local magnetic moments, spin-resolved electronic structure, and magnetic ordering, which are discussed separately. The presence of magnetism in these double perovskites originates primarily from the partially filled d orbitals of the transition-metal ions (Mo and W). Spin-polarized DOS, shown in Fig. 6, reveals a clear asymmetry between the up-spin and down-spin channels near the Fermi level, confirming the existence of localized magnetic moments associated with Mo-4*d* and W-5*d* states. In both materials, the major contribution to the magnetic moment arises from the transition-metal *d* orbitals, while Cs, K, and Br atoms contribute negligibly. The calculated local magnetic moments per formula unit are approximately 3 (µ_B_) for both compounds. These values represent the magnitude of the local spin polarization obtained from spin-polarized calculations initialized in the ferromagnetic (FM) configuration and indicate well-localized *d*-electron magnetism. It should be emphasized that an integer local magnetic moment does not, by itself, determine the magnetic ground state of the system. To identify the true magnetic ground state, total-energy calculations were performed for both ferromagnetic (FM) and antiferromagnetic (AFM) spin configurations. The magnetic phase stability was determined by comparing the total energies of these configurations after full self-consistent convergence. The results of these magnetic-phase optimizations are presented in Fig. 7. The total-energy comparison reveals that Cs_2_KMoBr_6_ stabilizes in an antiferromagnetic ground state, whereas Cs_2_KWBr_6_ favors a ferromagnetic ground state. In the AFM phase of Cs_2_KMoBr_6_, the local magnetic moments on equivalent Mo sites align antiparallel, leading to cancellation of the macroscopic magnetization, as expected for an antiferromagnet. Despite the presence of sizable local magnetic moments, the net magnetic moment of the AFM ground state is therefore zero. In contrast, Cs_2_KWBr_6_ exhibits parallel alignment of W-5*d* moments in the FM configuration, resulting in a nonzero net magnetization. These results demonstrate that while both compounds possess similar local magnetic moments, as listed in the Table 3, arising from transition-metal d states, their magnetic ordering differs due to differences in exchange interactions mediated by the Br ligands. Local magnetic moment is the spin density summed out in the atomic sphere of the transition metal, which is the spin state of the atom per se. On the other hand, when the spins are parallel, the summation of all the local and interstitial contributions in the whole unit cell gives the total magnetic moment, which represents the bulk magnetization. The local magnetic moment is the vector sum of these local magnetic moments in the AFM arrangement, and cancels to zero (or a small residual) because of the equal and opposite alignment of the spin sublattices^45^.

**Table 3:** The magnetic moments of Cs_2_KMoBr_6_ and Cs_2_KWBr_6_ perovskite materials.

Compounds	TM Ion	Local moment on TM (μB)	Total local moment per f.u (μB)*		Net magnetic moment of ground state (μB)	Predicted nature
Cs_2_KMoBr_6_	Mo	2.208	3.000	~ 3.0^43^	0.0	**AFM**
Cs_2_KWBr_6_	W	2.036	2.999		2.999	**FM**

*Total local moment per formula unit obtained from spin-polarized calculations initialized in the ferromagnetic configuration.

Furthermore, to confirm the magnetic phase of the materials analytically, their magnetic stabilization energy $\Delta E = E_{{{\mathrm{AFM}}}} - E_{{{\mathrm{FM}}}}$ (in Ry and meV) has been calculated and listed in Table 4. The negative value of the stabilization energy for Cs_2_KMoBr_6_ and its positive value for Cs_2_KWBr_6_ confirm their AFM and FM nature, respectively. The AFM ground state of Cs_2_KMoBr_6_ and the ferromagnetic ground state of Cs_2_KWBr_6_ highlight the sensitivity of magnetic ordering to transition-metal selection. This contrast in magnetic behavior may be relevant for spin-dependent electronic phenomena, although further studies of transport properties and spin dynamics are required to assess potential device applications^46–49^. While the predicted magnetic ordering and spin-dependent electronic structure suggest potential relevance for spintronic applications, further investigations involving transport properties and device-level modeling would be required to establish practical applicability.

**Table 4:** Magnetic ground state verification: Total energy and magnetic stabilization energy *Δ E* (in Ry and meV) for the FM and AFM phases of Cs_2_KXBr_6_ (X = Mo & W) materials.

Materials	E_FM_ (Ry)	E_AFM_ (Ry)	$\Delta \left( {Ry} \right) = E_{{{\mathrm{AFM}}}} - E_{{{\mathrm{FM}}}}$	$\Delta E\; \left( {{\mathrm{meV}}} \right) = E_{{{\mathrm{AFM}}}} - E_{{{\mathrm{FM}}}}$	Magnetic phase
Cs_2_KMoBr_6_	− 71,745.255	− 71,745.259	− 0.0041	− 55.78	**AFM**
Cs_2_KWBr_6_	− 95,978.278	− 95,978.269	+ 0.0085	+ 115.65	**FM**

### Mechanical properties

The fundamental parameters for evaluating mechanical stability and forecasting response to externally applied stresses are the elastic constants C_ij_ of crystalline materials. To characterize the elastic behavior in cubic systems, three independent constants, C_11_, C_12_, and C_44_, are needed^50^. The material’s resistance to uniaxial compressive strain along the primary axes is governed by C_11_, its resistance to lateral strain under uniaxial stress is reflected in C_12_, and C_44_ characterizes its stiffness against shear deformation. The Born criteria are used to thoroughly assess mechanical stability for cubic halide perovskite^6,51^. Which imposes the following conditions;

$$C_{11} - C_{12} > 0, C_{11} + 2C_{12 } > 0, C_{11} > 0, C_{44} > 0$$.

In the case of an external load, these inequalities ensure that the material retains its structural integrity, preventing the structure from permanently deforming or collapsing. The elastic stiffness matrices for the materials are computed and given below;

$${\mathbf{C}}_{{{\boldsymbol{ij}}}} \left( {{\mathrm{Cs}}_{2} {\mathrm{KMoBr}}_{6} } \right) = \left( {\begin{array}{*{20}c} {{ }45.90{ }} & {10.50{ }} & {10.51{ }} & {1.449} & {0.59} & { - 2.93} \\ {{ }10.50{ }} & {{ }45.73{ }} & {1.572{ }} & {2.564} & {0.81} & { - 2.79} \\ {{ }10.52{ }} & {1.572{ }} & {45.65{ }} & {1.311} & {0.089} & { - 2.122} \\ {{ }1.922{ }} & {3.964{ }} & {2.511} & {9.20} & {1.042} & { - 1.766} \\ {0.379} & {0.573{ }} & {0.289} & {0.41} & {9.20} & { - 1.193} \\ { - 2.93} & { - 1.79{ }} & { - 2.25} & { - 2.895} & {{ } - 1.193} & {9.20} \\ \end{array} } \right)$$

and;

$${\mathbf{C}}_{{{\boldsymbol{ij}}}} \left( {{\mathrm{Cs}}_{2} {\mathrm{KWBr}}_{6} } \right) = \left( {\begin{array}{*{20}c} {{ }47.64{ }} & {12.00{ }} & {12.08{ }} & { - 0.069} & { - 0.065} & {0.0212} \\ {{ }12.00{ }} & {47.71{ }} & {0.7017{ }} & { - 0.04958} & {0.0554} & {0.0351} \\ {{ }12.08{ }} & {12.85{ }} & {47.66} & { - 0.01555} & { - 0.013} & {0.0293} \\ {{ } - 0.15{ }} & { - 0.051{ }} & { - 0.0166} & {10.50} & { - 0.044} & {0.066} \\ { - 0.03} & {0.05545{ }} & { - 0.0127} & { - 0.03417} & {10.52} & {0.0018} \\ {0.051} & {0.042} & {0.0393} & {0.034} & {{ }0.004} & {10.53} \\ \end{array} } \right)$$

The structural stability of Cs_2_KXBr_6_ (X = Mo, W); HDPs is confirmed by the computed elastic constants C_11_, C_12_, and C_44_, which meet all Born conditions^52,53^. This compliance highlights the suitability for applications that rely on mechanical resilience, like high-pressure applications. The computed Bulk modulus (B), Young modulus (E) and Poisson’s ratio *ν* are defined mathematically as below^54^;

7 $$B = \frac{1}{3}\left( {2C_{12} + C_{11} } \right)$$

8 $$E = \frac{9GB}{{3B + G}}$$

9 $$\nu = \frac{3B - 2G}{{2\left( {3B + G} \right)}}$$

where $C_{12}$ and $C_{11}$ are elastic constants, and G is the shear modulus. The shear modulus (G) measures a material’s ability to withstand deformation when subjected to shear stress, which occurs due to forces applied tangentially to its surface. The G is calculated using the Hill averaging approach, specified as G = (G_V_ + G_R_)/2, where the symbols G_V_ (Voigt limit) and G_R_ (Reuss limit) represent the upper and lower bounds of the shear modulus, respectively, defined in terms of elastic constants as;

10 $$G_{\nu } = \frac{1}{5}\left( {3C_{44} - C_{12} + C_{11} } \right)$$

11 $$G_{R} = \frac{{5\left( {{\mathrm{C}}_{11} - {\mathrm{C}}_{12} } \right){\text{ C}}_{44} }}{{4{\mathrm{C}}_{44} + { }3\left( {{\mathrm{C}}_{11} - {\mathrm{C}}_{12} } \right)}}$$

**Fig. 8:**
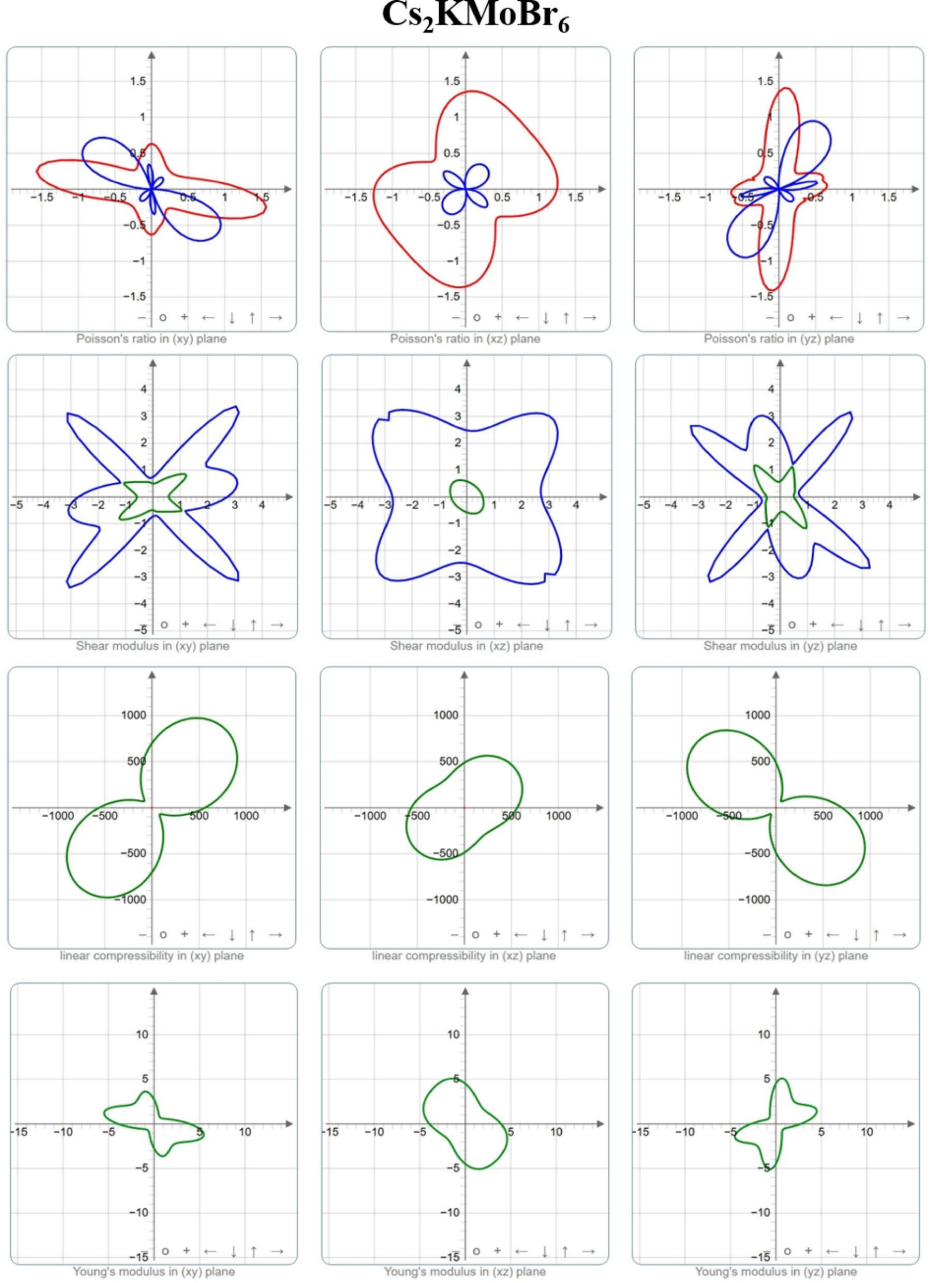

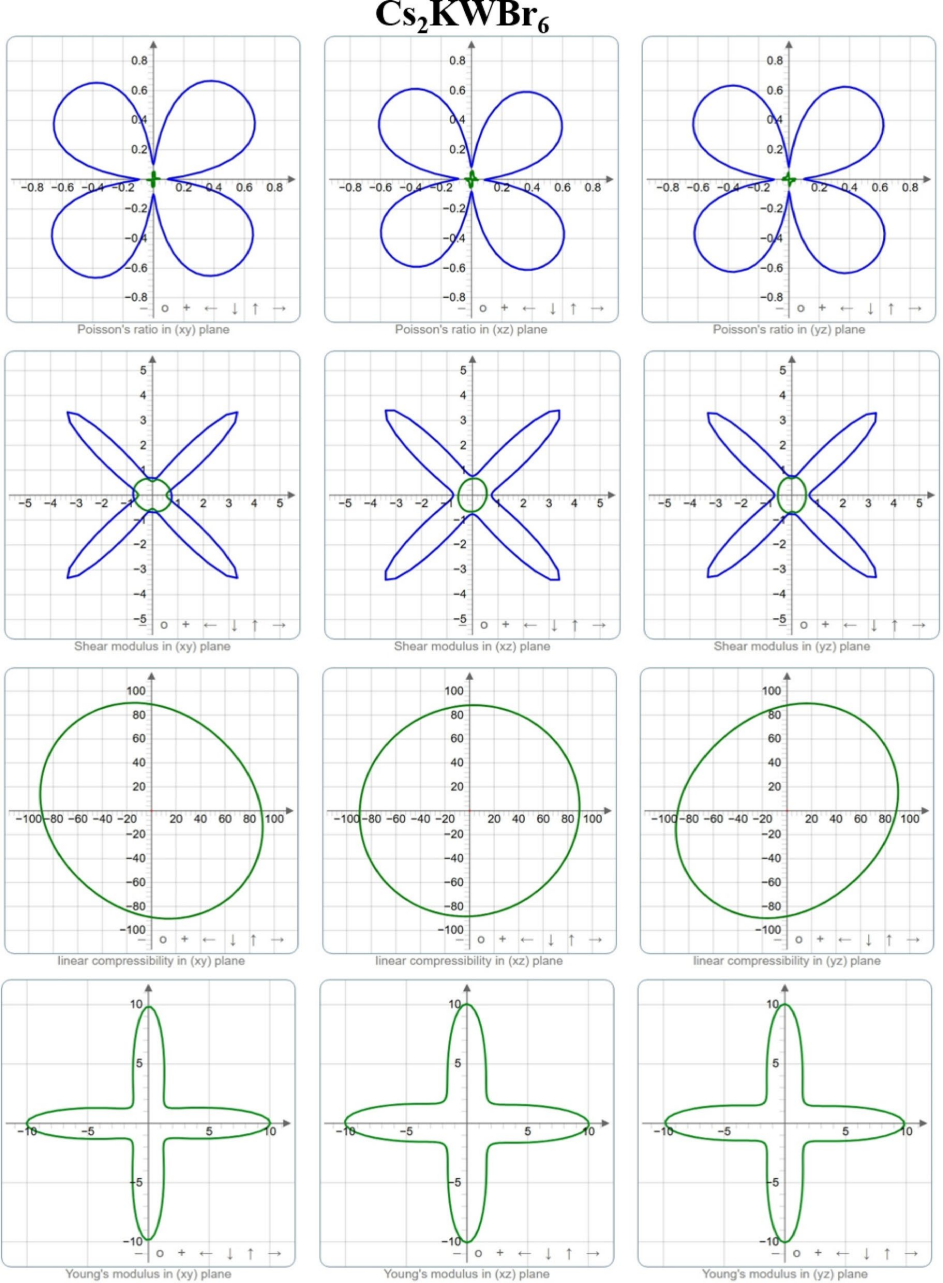
2D anisotropic illustrations of (**a**) Poisson’s, (**b**) Shear modulus, (**c**) Linear Compressibility, and (**d**) Young’s Modulus ratio of both materials.

The predicted values of these parameters are listed in the Table 5. Furthermore, 2D anisotropic visualizations of Poisson’s ratio, Shear modulus, linear Compressibility, and Young’s modulus of both materials are investigated and plotted in Fig. 8, which demonstrate the directional dependence of critical elastic parameters. The plot analysis indicates that the mechanical stability of Cs_2_KMoBr_6_ and Cs_2_KWBr_6_ differs significantly when the elastic anisotropy is compared. Although the shear and Young’s moduli of both double perovskites show directional dependence, Cs_2_KWBr_6_ exhibits superior structural stability, as displayed by perfectly isotropic linear compressibility and identical elastic behavior across all principal planes, which is consistent with a stable cubic symmetry. On the other hand, Cs_2_KMoBr_6_ exhibits significant anisotropy in linear compressibility and fluctuating planar projections, which are signs of a lattice distortion with reduced symmetry. As a result, it is anticipated that Cs_2_KWBr_6_ will be more mechanically resilient under hydrostatic pressure, while the Cs_2_KMoBr_6_ variant’s anisotropic compressibility could cause differential strain and make it more vulnerable to micro-cracking during processing or thermal cycling.

**Table 5:** The bulk modulus, Young’s modulus, shear modulus, and Poisson’s ratio values of both materials.

Mechanical properties	Cs_2_KMoBr_6_	Cs_2_KWBr_6_
Bulk modulus (B)	22.30 GPa	23.88 GPa
Shear modulus (G)	12.07 GPa	12.91 GPa
Young’s modulus (E)	30.77 GPa	33.09 GPa
Poisson’s ratio (ν)	0.274	0.282

### Optical properties

**Fig. 9:**
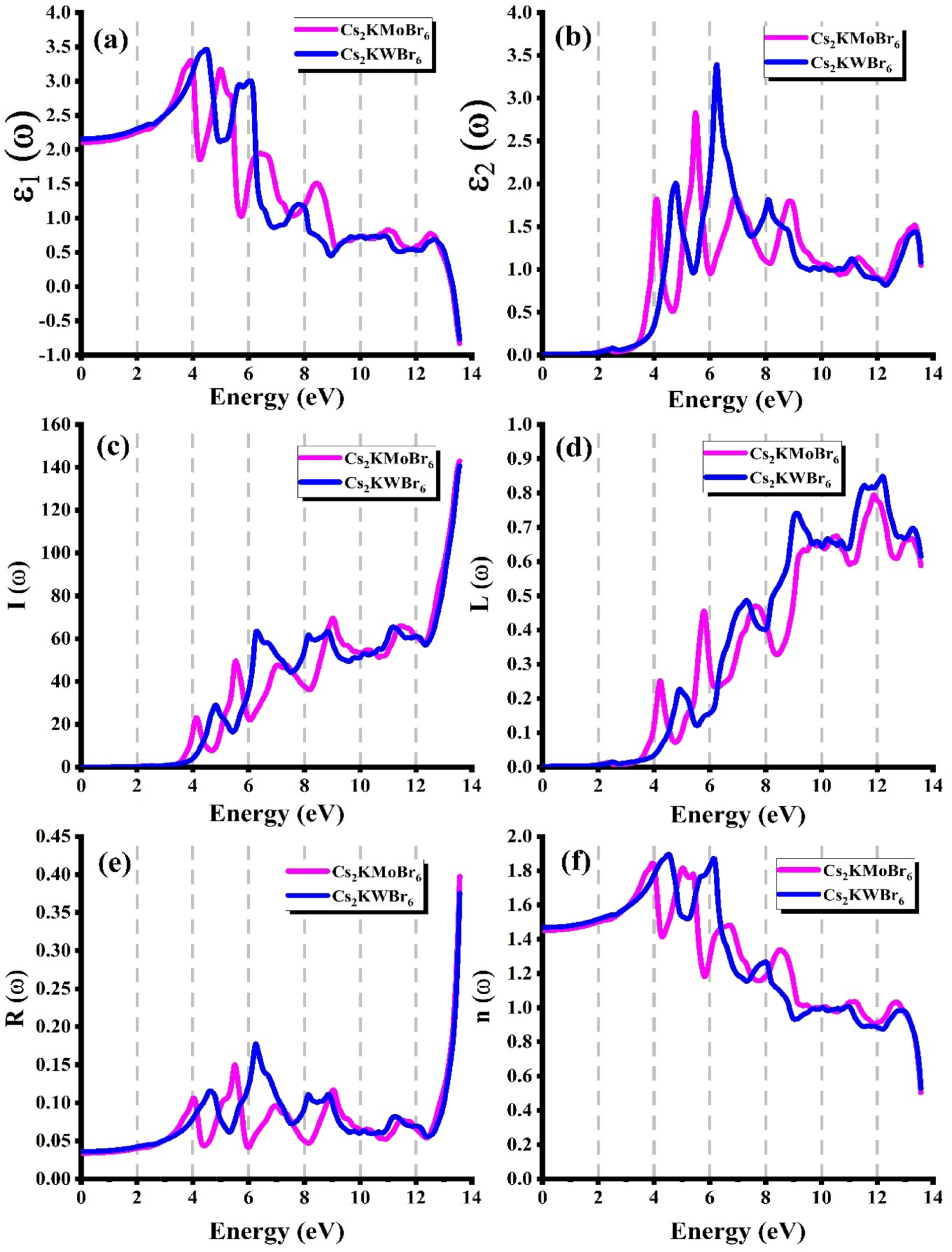
The optical parameters; (**a**) real part; ε_1_(ω), (**b**) imaginary part; ε_2_(ω), (**c**) absorption coefficient; I (ω), (**d**) energy loss function; L (ω), (**e**) reflectivity coefficient; R (ω) and (**f**) refractive index n (ω) of materials.

The optical properties of materials are investigated to understand their interaction with electromagnetic radiation and to assess their potential relevance for optoelectronic and energy-related applications. The optical characteristics offer a remarkable and comprehensive method for analyzing the band structure, lattice oscillations, and various impurity levels^55^. These properties include the real (ε_1_(ω)) and imaginary (ε_2_(ω)) parts of the dielectric function, the absorption coefficient I(ω), and several others have been computed, in up-spin, from 0 to 14 eV energy range, and plotted in Fig. 9(a–f). The dielectric functions explain the polarization and absorption of electromagnetic radiation in a particular material. The degree of light absorption is represented by the imaginary portion of the dielectric function, ε_2_(ω), whereas the real part, ε_1_(ω), indicates the polarization response of the material to the incident electromagnetic field and governs its dispersive optical behavior. In optoelectronic devices, the absorption and emission of light intensities are determined by the strength of the transitions between and within bands. The Fig. 9(a) displays the computed real parts of the function of both materials. The static real part ε_1_(0) is an important parameter that represents the electronic part of the function, having values of 2.1 and 2.2 for Cs_2_KMoBr_6_ and Cs_2_KWBr_6_ materials, respectively. The Penn’s model^56^, which expresses the inverse relation between the static real parts and the energy band gaps of the materials, is found to be valid for both materials. The plots clearly show that ε_1_(ω) initially rises and reaches its maximum value at 4.2 eV and 3.9 eV for Cs_2_KMoBr_6_ and Cs_2_KWBr_6_, respectively. The Fig. 9(b) displays the imaginary part ε_2_(ω), which describes the interband transitions of electrons. The plots show that the ε_2_(ω), for Cs_2_KMoBr_6_ and Cs_2_KWBr_6_ materials, have threshold values of 1.8 eV and 1.7 eV, respectively, which correspond to the energy band gap values of the materials. The ε_2_ (ω) values of Cs_2_KMoBr_6_ and Cs_2_KWBr_6_ start increasing beyond the threshold values, and reach a maximum value at 5.2 eV and 6.1 eV, respectively. The electronic interband transitions behind the maximum optical absorption can be understood by DOS analysis (see Fig. 6). The DOS plots indicate that the maximum absorption for Cs_2_KMoBr_6_, in up-spin, is caused by the electronic transitions from Mo-4*d* of the valence band to Mo-4*d* of the conduction band and in down-spin, from Br-4*p* of the valence band to the Mo-4*d* of the conduction band. For Cs_2_KWBr_6_, the up-spin is caused by the electronic transitions from W-5*d* of the valence band to the W-5*d* of the conduction band, and in down-spin, from Br-4*p* of the valence band to the W-5*d* of the conduction band. There are several other peaks of ε_2_ (ω); each peak also represents the interband transitions of electrons. These peaks are observed to be, for Cs_2_KMoBr_6_, at 3.6 eV, 6.5 eV, and 8.7 eV, and for Cs_2_KWBr_6_, located at 4.3 eV and 8 eV energies. The absorption coefficient I (ω) of the materials, displayed in Fig. 9(c), indicates that the materials have specific threshold values of absorption, which confirms the energy band gaps of the materials. The optical absorption starts beyond the threshold values and increases with the increase of incident photon energies. The plots clearly indicate that maximum absorption takes place at 5.5 eV and 6.3 eV for Cs_2_KMoBr_6_ and Cs_2_KWBr_6_ materials, respectively. The optical absorption energy ranges suggest that the materials are promising for solar cells and higher energy-absorbing applications^57^. The electron energy loss function L (ω), plotted in Fig. 9(d), indicates the low energy loss in the visible region and increases with the rise of incident photon energies, reaching its maximum value of 13.5 eV, for both materials. The surface morphology of the materials can be analyzed by the reflectivity coefficient, R (ω), plotted in Fig. 9(e). The static reflectivity R (0) defines how a substance interacts with low-energy electromagnetic radiations like radio waves or static fields. The static reflectivity of Cs_2_KMoBr_6_ and Cs_2_KWBr_6_ is 0.026 (2.6%) and 0.025 (2.5%), respectively. The refractive index n(ω) of materials is depicted in Fig. 9(f), which characterizes the bonding nature of that substance. The static values of refractive index n (0) are 1.2 and 1.4 for Cs_2_KMoBr_6_ and Cs_2_KWBr_6_, respectively. The n (ω) rises with the rise of photon energy and reaches its maximum values at 4 eV and 3.5 eV for Cs_2_KMoBr_6_ and Cs_2_KWBr_6_, respectively. The calculated optical response, including absorption and refractive index, indicates that these materials interact efficiently with electromagnetic radiation in the visible to ultraviolet range. These characteristics suggest potential relevance for optoelectronic applications such as photodetectors or energy-harvesting systems. However, it should be noted that practical device performance depends on additional factors such as carrier mobility, defect tolerance, and interface properties, which are beyond the scope of the present study^54,58–62^.

## The conclusion

The present study provides new insight into the role of A-site substitution and transition-metal selection in halide double perovskites. In contrast to previously reported Na- and Li-based systems, the incorporation of K modifies the structural and electronic environment, leading to distinct electronic bandwidths and magnetic interactions. Furthermore, the comparison between Mo (4*d*) and W (5*d*) compounds reveals how orbital extension and hybridization strength govern spin-dependent band gaps and magnetic ordering. These findings highlight a clear structure–property relationship and demonstrate that targeted compositional tuning can be used to design double perovskites with tailored optoelectronic and spintronic properties. The spin-based ab initio investigations of Cs_2_KXBr_6_ (X = Mo & W); HDPs, are conducted to study their structural, electronic, and magneto-optic characteristics for many technological applications. The FP-LAPW method incorporated in the Wein2K code has been employed to compute the material’s characteristics. The exchange–correlation potential TB-mBJ has been utilized to achieve the accurate band gaps of the materials. The absence of imaginary frequency in the phonon dispersion spectrum and small energy fluctuations in AIMD plots indicate the dynamical stability of the materials. The negative formation energies of the materials confirm their thermodynamic stability. The electronic nature of the materials has been predicted by studying their electronic band structures and density of states. The electronic band structures, in both spin configurations, of both materials indicate the semiconducting nature of both materials. The energy band gaps of Cs_2_KMoBr_6_ are 1.7 eV (up-spin) and 2.8 eV (down-spin), and Cs_2_KWBr_6_ are 1.6 eV (up-spin) and 3.3 eV (down-spin). The energy band gaps are verified by the DOS of both materials. The major contribution to the electronic conductivity, in up-spin, of Cs_2_KMoBr_6_ is due to K-d and Mo-d states, and in Cs_2_KWBr_6_, the contribution is due to W-*d* states of the material. The DOS predicts the magnetic nature of the materials. The computed total magnetic moments of both materials are 3 (µ_B_). The optimizations in magnetic phases predict that Cs_2_KMoBr_6_ is anti-ferromagnetic while Cs_2_KWBr_6_ is ferromagnetic. These materials are promising for spintronic and magnetic data storage applications. The optical characteristics are investigated in the 0–14 eV energy range to analyze the interaction of the material with electromagnetic radiation and to explore its potential relevance for optoelectronic and energy-related applications. The results indicate that Cs_2_KXBr_6_ (X = Mo, W) materials exhibit stable structural, electronic, and magnetic properties within the framework of first-principles calculations. The combination of semiconducting behavior and spin-dependent electronic structure suggests potential relevance for optoelectronic and spin-dependent applications. However, further experimental validation and device-level investigations are necessary to fully evaluate their practical performance. It is important to note that first-principles calculations provide insight into intrinsic material properties under ideal conditions; real-device performance may be influenced by defects, interfaces, and external conditions not considered in this work.

## Data Availability

The datasets generated during and/or analyzed during the current study are available from the corresponding author on reasonable request.
